# Resting energy expenditure is not influenced by classical music

**DOI:** 10.1186/1477-5751-4-6

**Published:** 2005-08-31

**Authors:** Ebba Carlsson, Hannah Helgegren, Frode Slinde

**Affiliations:** 1 Dept. of Clinical Nutrition, P. O Box 459, Sahlgrenska Academy at Göteborg University, SE-405 30 Göteborg, Sweden

## Abstract

Obesity shows an increasing prevalence worldwide and a decrease in energy expenditure has been suggested to be one of the risk factors for developing obesity. An increase in resting energy expenditure would have a great impact on total energy expenditure. This study shows that classical music do not influence resting energy expenditure compared to complete silence. Further studies should be performed including other genres of music and other types of stress-inductors than music.

## Findings

Obesity shows an increasing prevalence worldwide [1] and a decrease in energy expenditure has been suggested to be one of the risk factors for developing obesity [2]. Increasing energy expenditure could be done by increasing physical activity, but resting energy expenditure (REE) is the largest part of an humans' energy expenditure (70–80%), and an increase in REE would have a large impact on total energy expenditure. REE is assessed by indirect calorimetry by measurements of oxygen consumption and carbon dioxide production which, when known, is calculated into energy expenditure [3]. It is known that ingestion of food increases resting energy expenditure – also called diet induced thermogenesis [4] Nicotine and caffeine have also been shown to increase energy expenditure [5]. None has however studied the effect of external sound stimuli, such as music, on REE. The aim of the current study was to assess if classical music has an effect on REE, and if there are differences between different types of classical music.

In this randomized cross-over study, 2 different music CD's were used. Both CD's started with 10 minutes of silence and were followed by 10 minutes of calm classical music and 10 minutes of stressful classical music, presented in Table 1. The order of music differed between the two CD's, which was randomly chosen for each subject. Classical music was chosen for both stressful and calm music to limit confounding effects from the subjects' taste of music. A pre-study power-calculation showed that to be able to detect a statistical significant (p < 0.05) difference at 420 kJ/day (judged as clinical relevant) with a power of 80%, 40 subjects should be included. To allow for drop-out, 43 healthy volunteers (31 women and 12 men) were included, all participants gave written informed consent. Following measurement of height and weight, REE was measured by indirect calorimetry using a ventilated hood system, the Deltatrac™ II Metabolic Monitor (Datex, Helsinki, Finland). Before each measurement, the equipment was calibrated with gas mixtures of known O_2 _and CO_2 _contents according to the instructions from the manufacturer. The subjects were instructed to limit their physical activity the evening before measurement. All subjects were measured after an overnight fast and they arrived from their home by car or public transport. After 30 minutes rest in the supine position REE was measured during 35 minutes when the subjects were awake. Due to adaptation to the inside-hood environment, the first five minutes were eliminated from the total result. The music was provided through earphones and measurements were performed in an environmental temperature of 20–24°C. After completion of the measurement, the subjects were asked how they perceived each part of the music, as calm or stressful, or something else. Data are presented as mean and standard deviation. To compare REE during silence to the calm and stressful music, two-sided paired Student's *t*-tests were performed.

**Table 1:** Description of the calm and stressful music which each lasted for 10 minutes

**Calm music**		**Stressful music **	
*Composer*	*Piece of music*	*Composer*	*Piece of music*
Erik Satie	Gymnopédie No 1	Béla Bartók	String quartet No 4 prestissimo con sordino
Erik Satie	Gymnopédie No 3	Igor Stravinsky	From The Fire Bird: "Infernal dance of all Kashcers's subjects"
Johann Sebastian Bach	Air	Hans Werner Henze	2^nd ^movement "Dies irae" from Requiem for piano, trumpet and chamber orchestra

Forty subjects, 29 women and 11 men, completed the study. One subject dropped out because of feeling uncomfortable in the ventilated hood, one subject due to technical issues with the indirect calorimeter, and one subject due to problems with the CD-player. Mean (SD) age of the subjects were 35 (14) y, body height 172 (10) cm, body weight 68 (13) kg, and body mass index 23 (3) kg/m^2^. Mean (SD) REE during silence was 5720 (1063) kJ/day. No significant differences in REE between silence and the two sets of music were found, 5710 (1054) kJ/day during calm music (p = 0.57) and 5740 (1046) kJ/day during stressful music (p = 0.43). Thirty-eight subjects perceived the calm music as calm and 28 subjects the stressful music as stressful. However, analyzing the results regarding to their own perception of the results, did not yield any statistically significant differences in measured REE between silence and the two music periods.

This study could not detect any statistical significant or clinical relevant influences of music on REE, and then theoretically not on total energy expenditure. We chose to compare classical calm music to classical stressful music. This was to limit the confounding effect of the subjects own music preferences. When the stressful music was selected, not only tempo of the music was taken into consideration. The stressful music was also supposed to be irregular, have large differences between high and low frequencies, include many abrupt sounds, and give a sense of unpredictability. Most of the subjects perceived the calm music as calm and the stressful music as stressful, even if some subjects experienced the stressful music as "other". Maybe the stressful music was not stressful enough. Further studies should be conducted to investigate other types of music, i.e. pop music vs. heavy metal, and preferably also other types of stress-inductors than music combined with measurements of heart rate and other measures of stress. The results from this study do not support that music during rest could be used in obesity prevention or treatment alone, but music could of course be combined with physical activity to achieve an increase in total energy expenditure.

## List of abbreviations

REE – resting energy expenditure

CD – compact disc

SD – standard deviation

## Authors' contributions

EC and HH participated in the study design, carried out the data collection and analyzed the results. FS conceived the study, and participated in its design and coordination and drafted the manuscript. All authors read and approved the final manuscript.
